# Retraction: Ubiquitin/SUMO Modification Regulates VHL Protein Stability and Nucleocytoplasmic Localization

**DOI:** 10.1371/journal.pone.0265155

**Published:** 2022-03-04

**Authors:** The PLOS ONE Editors

Following the publication of this article [1], concerns were raised regarding results presented in Figs 3, 4, and 5. Specifically,

- In Fig 3D, the band in the first lane of the SP1 panel appears similar to the band in the first lane of the Actin panel when flipped and rotated.
- Irregularities have been detected in the following panels:
      ○ Fig 4C, IP/IB FLAG panel and relB HA panel.
      ○ Fig 5A, FLAG-VHL HIF2α panel.
- The first, second, and third lane of the Fig 4C WCL panel appear similar.

The authors have provided individual level data underlying the graphs and most blots presented in Figs 1–5. The underlying blots provided for Fig 3D confirm that despite their similarity, the bands are not identical. Furthermore, the authors confirm that the blot underlying the Fig 5A FLAG-VHL HIF2α panel, has been spliced, and that the results in the published panel do not align correctly with the labels.

The underlying data provided for the Fig 4C IP/IB: FLAG and WCL panels confirm that these panels have been prepared using spliced blots: for the IP/IB FLAG blot, lanes 1 and 4–7 are from a different blot than lanes 2–3, and for the WCL blot, lanes 1–3 are from a different blot than lanes 4–7. The authors explained the blots were spliced as there was not sufficient space to run al samples on one gel. Since the control and experimental lanes in the WCL and IP/IB: FLAG panels were obtained on different blots, they cannot be compared directly and hence the result and conclusions statements based on Fig 4C are not supported.

Images provided in support of Fig 4C did not resolve the concern about similarities between lanes of the WCL panel, and raised additional data reporting concerns. The image data provided for lanes 1–3 of the WCL blot were not an exact match with the published panel, and included neither size markers nor controls needed to verify the negative results. The underlying data provided for the IP/IB FLAG blot showed additional bands in lanes 2–7 just below the 34 kDa marker which were not shown in the published panel. The authors explained that these bands were inadvertently excluded during panel preparation.

In light of the unresolved concerns about image data reporting and the reliability of results in Fig 4C, the *PLOS ONE* Editors retract this article.

ESR and QC did not agree with the retraction.

## References

[1] Cai Q, Robertson ES (2010) Ubiquitin/SUMO Modification Regulates VHL Protein Stability and Nucleocytoplasmic Localization. PLoS ONE 5(9): e12636. 10.1371/journal.pone.0012636 20844582 PMC2936558

